# GDF3 Protects Mice against Sepsis-Induced Cardiac Dysfunction and Mortality by Suppression of Macrophage Pro-Inflammatory Phenotype

**DOI:** 10.3390/cells9010120

**Published:** 2020-01-03

**Authors:** Lu Wang, Yutian Li, Xiaohong Wang, Peng Wang, Kobina Essandoh, Shunan Cui, Wei Huang, Xingjiang Mu, Zhenling Liu, Yigang Wang, Tianqing Peng, Guo-Chang Fan

**Affiliations:** 1 Department of Critical Care Medicine, Renmin Hospital of Wuhan University, Wuhan 430060, China; wanglu@whu.edu.cn; 2 Department of Pharmacology and Systems Physiology, University of Cincinnati College of Medicine, Cincinnati, OH 45267, USA; li3yt@mail.uc.edu (Y.L.); wang2xn@ucmail.uc.edu (X.W.); wang2pe@ucmail.uc.edu (P.W.); essandkq@mail.uc.edu (K.E.); cuisa@ucmail.uc.edu (S.C.); muxg@ucmail.uc.edu (X.M.); liuzl@mail.uc.edu (Z.L.); 3 Department of Critical Care Medicine, Shandong Provincial Hospital Affiliated to Shandong University, Jinan 250021, China; 4 Department of Pharmacology, University of Michigan Medical School, Ann Arbor, MI 48109, USA; 5 Institute of Anesthesiology and Critical Care Medicine, Union Hospital, Tongji Medical College, Huazhong University of Science and Technology, Wuhan 430020, China; 6 Department of Pathology and Laboratory Medicine, University of Cincinnati College of Medicine, Cincinnati, OH 45267, USA; huangwe@ucmail.uc.edu (W.H.); yi-gang.wang@uc.edu (Y.W.); 7 Critical Illness Research, Lawson Health Research Institute, London, ON N6A 4V2, Canada; tpeng2@uwo.ca

**Keywords:** growth differentiation factor 3, endotoxin, cardiac dysfunction, cardiac inflammation, Smad, macrophage polarization, NLRP3, sepsis biomarker

## Abstract

Macrophages are critical for regulation of inflammatory response during endotoxemia and septic shock. However, the mediators underlying their regulatory function remain obscure. Growth differentiation factor 3 (GDF3), a member of transforming growth factor beta (TGF-β) superfamily, has been implicated in inflammatory response. Nonetheless, the role of GDF3 in macrophage-regulated endotoxemia/sepsis is unknown. Here, we show that serum GDF3 levels in septic patients are elevated and strongly correlate with severity of sepsis and 28-day mortality. Interestingly, macrophages treated with recombinant GDF3 protein (rGDF3) exhibit greatly reduced production of pro-inflammatory cytokines, comparing to controls upon endotoxin challenge. Moreover, acute administration of rGDF3 to endotoxin-treated mice suppresses macrophage infiltration to the heart, attenuates systemic and cardiac inflammation with less pro-inflammatory macrophages (M1) and more anti-inflammatory macrophages (M2), as well as prolongs mouse survival. Mechanistically, GDF3 is able to activate Smad2/Smad3 phosphorylation, and consequently inhibits the expression of nod-like receptor protein-3 (NLRP3) in macrophages. Accordingly, blockade of Smad2/Smad3 phosphorylation with SB431542 significantly offsets rGDF3-mediated anti-inflammatory effects. Taken together, this study uncovers that GDF3, as a novel sepsis-associated factor, may have a dual role in the pathophysiology of sepsis. Acute administration of rGDF3 into endotoxic shock mice could increase survival outcome and improve cardiac function through anti-inflammatory response by suppression of M1 macrophage phenotype. However, constitutive high levels of GDF3 in human sepsis patients are associated with lethality, suggesting that GDF3 may promote macrophage polarization toward M2 phenotype which could lead to immunosuppression.

## 1. Introduction

Sepsis is a life-threatening syndrome following a dysregulated host response to infection with an increasing incidence and nearly 40% of mortality, as defined by the Sepsis-3 definition [1,2]. Sepsis is a highly coordinated process with sequential events including immune response, clearance of bacteria or endotoxins, and multiple tissue remolding. Importantly, it is well accepted that increased systemic levels of endotoxins during sepsis is responsible for direct activation of innate immune cells (i.e., macrophages), leading to the production of inflammatory mediators and cytokines [3]. As a matter of fact, the long-term effects of subclinical endotoxemia are associated with the risk of multiple organ failure, of which cardiac dysfunction has been linked with increased risk of mortality and morbidity in endotoxemic patients [4]. Despite decades of intensive study, the precise mechanisms underlying the endotoxin-induced inflammation and myocardial depression remain obscure.

During sepsis, macrophages act as the main players in the first-line defense against pathogens and modulate inflammatory response and tissue homeostasis [5]. Recent studies have indicated that macrophages are not homogenous, and exist along with subpopulations of pro-inflammatory (M1) and tissue repairing (M2) phenotypes, or mixture of both phenotypes [6]. The transition of macrophage function is dependent upon environmental signals. For example, M1 macrophages are antigen-presenting cells that can be induced by LPS or IFN-γ. They are mainly responsible for producing pro-inflammatory mediators like tumor necrosis factor-α (TNF-α), interleukin 6 (IL-6), interleukin 1β (IL-1β), monocyte chemoattractant protein-1 (MCP-1), and iNOS [6,7]. During early phase of sepsis, numbers of macrophages with M1 phenotype rise significant with primary function of killing bacteria [7]. On the flip side, M2 macrophages could be induced by IL-4, they are recognized by anti-inflammatory and tissue-repairing characteristics at later stage of sepsis [6]. These macrophages express high levels of markers, i.e., transforming growth factor β (TGF-β), Arg-1, CD206 and IL-10 [6]. Accordingly, this delicate balance of macrophage polarization ensures efficient toxin clearance and tissue repair. Therefore, it will be of great clinical interest to better understand how macrophage polarization is modulated, which can provide guidance to formulate personalized treatment based on patients’ macrophage profile.

Growth differentiation factor 3 (GDF3), a member of TGF-β superfamily, also referred to as Vgr-2, was initially identified to modulate early embryonic development, adipose-tissue homeostasis and energy balance by interacting with cell membrane activin receptor-like kinase type I receptor B (ACVR1B, ALK4) and ACVR1C (ALK7) [8,9]. Interestingly, several recent studies have suggested that GDF3 may play a critical role in modulating macrophage functions. For example, Varga et al reported that GDF3 could serve as an exclusively macrophage-derived factor in the restoration of skeletal muscle integrity [10]. Patsalos et al further observed that tissue-repairing macrophages could secret GDF3, which was markedly downregulated in the injured muscle of old mice relative to young mice [11]. Furthermore, GDF3 has been demonstrated to be an essential mediator for adipose tissue macrophage (ATM)-mediated fat metabolism [12]. In addition, a recent study by Camell et al suggests that the NLRP3 inflammasome may correlate with GDF3 in adipose tissue macrophages [13]. Nevertheless, the role of GDF3 in macrophage-regulated endotoxemia/sepsis remains unclear.

In light of the aforementioned findings, we hypothesized that GDF3 might be a beneficial factor for survival and cardiac function in septic patients and mouse models through skewing macrophage towards M2 phenotype. To test this hypothesis, we first determined GDF3 levels in sera collected from septic patient and healthy donors. Using both in vitro bone marrow-derived macrophages (BMDMs) and in vivo mouse model treated with recombinant GDF3 protein (rGDF3), we conducted a series of analyses to assess macrophage phenotype, systemic/cardiac inflammation and function, as well as animal survival after endotoxin LPS challenge. Lastly, the underlying mechanism was examined. Our study presented here defines a previously unrecognized role of GDF3 in macrophage polarization and endotoxin/sepsis-induced cardiac injury.

## 2. Materials and Methods

### 2.1. Human Patients and Controls

A total of 30 adult patients diagnosed with sepsis by clinical and laboratory investigations (15 were sepsis, 15 were septic shock) and 15 healthy donators were recruited. All patients were admitted or evaluated at follow up in Intensive Care Unit (ICU), Renmin Hospital of Wuhan University during the period from April to October 2018. Subjects in the control group were healthy donators who were recruited in the Medical Examination Center. Patients were diagnosed according to Sepsis-3 criteria [1]. Exclusion criteria were diagnosed with chronic heart, kidney, liver dysfunction, autoimmune diseases, malignancy cancers, or lack of tests outlined in the study. At the time of the ICU admission, blood samples were collected to measure liver function, kidney function, inflammatory cytokines and GDF3, then clinical data were recorded. The characteristics of the study groups are described in Supplement Table S1.

### 2.2. Animals

Wild-type (WT) mice (C57BL/6) were maintained and bred in the Division of Laboratory Animal Resources at the University of Cincinnati Medical Center. All animal experiments were performed based on the Guidelines for the Care and Use of Laboratory Animals prepared by the National Academy of Sciences, and approved by the University of Cincinnati Animal Care and Use Committee. 8–10-week-old male WT mice were used in this project.

### 2.3. Endotoxin-Induced Septic Mouse Model

Endotoxemia was induced in mice by intraperitoneal (IP) injection of LPS (10 mg/kg body weight, Sigma, St. Louis, MO, USA) and same volume of PBS was injected as controls. A single dose of recombinant mouse GDF3 protein (rGDF3, dissolved in PBS buffer, 10 μg/kg body weight) or control bovine serum albumin (BSA) was injected intraperitoneally into mice 12 h before LPS challenge. Recombinant mouse GDF3 protein (R&D Systems, Minneapolis, MN, USA) was added with BSA as a carrier protein to enhance protein stability and increase shelf-life. Endotoxemic mice and their corresponding controls were sacrificed after LPS injection at different time points. Sera were collected for ELISA measurements, heart tissues were collected for isolation of macrophages and flow cytometry analysis. Heart function of another cohort of mice were measured by transthoracic echocardiography. The survival rate was monitored every 4–6 h for 72 h.

### 2.4. Macrophages

Bone marrow-derived macrophages (BMDMs) were isolated from femoral and tibia of C57BL/6 mice and were cultured/differentiated into macrophages according to the protocol as described previously [14]. In brief, BMDMs were obtained by flushing the tibia and femur with Dulbecco’s modified Eagle medium (DMEM) supplemented with 10% fetal bovine serum (FBS), filtered through 70 µm cell strainer Cells were then pelleted and washed with PBS. Bone marrow cells were cultured in 10mL of DMEM supplemented with 10% FBS, glutamine, 10% L929 cell supernatant and 100 U/mL penicillin and 100 μg/mL streptomycin at a density of 10^6^ cells/mL in dishes at 37 °C and humidified 5% CO_2_ for 7 days to ensure complete differentiation to macrophages (BMDMs). Cells were then harvested with cold PBS, washed, scraped and suspended in DMEM supplemented with 10% FBS, and used at a density of 2–5 × 10^5^ cells/mL. RAW264.7 cells (mouse macrophage cell line) were cultured in DMEM supplemented with 10% FBS, glutamine and 100 U/mL penicillin and 100 μg/mL streptomycin. To determine the mechanism underlying the GDF3-mediated action, RAW264.7 cells were incubated with SB431542 (10 μM, S4317, Sigma-Aldrich), an inhibitor of ALK4/5/7, upstream kinases of Smad2/3, for 30 min, followed by the addition of rGDF3 (50 ng/mL) for 1 h, then LPS (10 ng/mL) was added for 12 h. The supernatants were collected for cytokines measurements and total RNA were extracted from cells for real-time quantitative PCR (RT-PCR) analysis.

### 2.5. Real-Time Quantitative PCR Analysis

Total RNA was extracted from different tissues of WT mice or BMDMs by the miRNeasy Mini Kit (217004, Qiagen, Hilden, Germany), blood RNA was extracted by PureLink Total RNA Blood Purification Kit (K156001, Invitrogen, Carlsbad, CA, USA). The quantity and quality of the RNA were determined using the NanoDrop 2000 system (Thermo Fisher Scientific Inc., Waltham, MA, USA). Complementary DNA was generated by miScript PCR Starter kit (Qiagen). Then real-time quantitative PCR was performed in duplicate for each sample using SYBR Green ER qPCR SuperMix (Invitrogen) and StepOne Real-Time PCR System (Thermo Fisher Scientific Inc.). Gene expression was normalized to the housekeeping gene GAPDH or 18S and calculated by the 2^−ΔΔCt^ method. The primer sequences for RT-PCR are shown in Supplemental Table S2.

### 2.6. Flow Cytometry Analysis of BMDMs and Cardiac Macrophages

Flow cytometry analysis was performed by the standard method, as previously described [15,16] via an LSR II flow cytometer unit (BD Biosciences, San Jose, CA, USA). Briefly, mice were euthanized and perfused with 10 mL PBS via left ventricle to remove circulating immune cells; then heart tissue was isolated and minced into small pieces, followed by digestion with HBSS with Collagenase IV (2 mg/mL, #LS004188, Worthington Biochemical Co., Lakewood, NJ, USA), Dispase II (1.2 U/mL, #D4693, Sigma) and 0.9 mM CaCl_2_, then incubated at 37 °C for 45 min with gentle agitation. Next, tissue was filtered through 40 µm cell strainer and centrifuged at 500× *g* at 4 °C for 5 min. The pellet was re-suspended in flow cytometry soring buffer (HBSS with 1 mM EDTA, 25 mM HEPES and 1% FBS). After blocking with CD16/32 antibody, BMDMs and macrophages collected from mouse hearts (above) were surface-stained with antibodies against CD11b (47-0112-80, eBioScience, San Diego, CA, USA), CD45.2 (109816, Bio-Legend, San Diego, CA, USA), CD206 (MCA2235 A700, Bio-Rad, Hercules, CA, USA), F4/80 (123135, BioLegend), Ly6C (17-5932-82, eBioScience), Ly6G (127628, Bio-Legend), MHC-II (46-5321-82, eBioScience). The data were analyzed using FCSexpress software (ACEA Biosciences, San Diego, CA, USA).

### 2.7. Transthoracic Echocardiography for Measurement of Cardiac Function

Mouse cardiac function was determined by transthoracic echocardiography using preclinical ultrasound system (Vevo 2100, FUJIFILM Visual Sonics, Toronto, Canada) with a 30 MHz linear array transducer. Left ventricular (LV) end-systolic inner diameter (LVIDs), and LV end-diastolic inner diameter (LVIDd) were measured from M-mode recordings. LV ejection fraction (EF) was calculated as: EF (%) = [(LVIDd)^3^ − (LVIDs)^3^/(LVIDd)^3^] × 100. LV fractional shortening (FS) was calculated as [(LVIDd − LVIDs)/LVIDd] × 100.

### 2.8. Western Blot Analysis

Cultured macrophages were collected and lysed in NP-40 lysis buffer (FNN0021, Invitrogen) containing 0.1 mM PMSF, protease inhibitor cocktail (Roche) and phosphatase inhibitor cocktail (CST, 5870s). Protein concentrations were then measured by BCA protein assay method. Protein samples (20–50μg) were loaded to 10–12% SDS-PAGE and then transferred to 0.2 μm nitrocellulose blotting membrane (GE Healthcare Life Science, Chicago, IL, USA), followed by incubation with 5% non-fat milk blocking buffer for 1 h. Membranes were then incubated with the following primary antibodies at 4 °C overnight: against p-SMAD2/3 (Cell Signaling Technology, 8828, 1:1000), SMAD2/3 (AF3797, R&D Systems, 1:1000), NLRP3 (AG-20B-0014, AdipoGen, San Diego, CA, USA, 1:330), GAPDH (2118S, Cell Signaling Technology, Danvers, MA, USA, 1:2000). Membranes were incubated with corresponding HRP-conjugated secondary antibody for 1.5 h and visualized by ECL kits (Pierce, Rockford, IL, USA). The levels of protein were quantified by densitometry and the intensity of the GAPDH band was used as a loading control for comparison between samples.

### 2.9. Enzyme-Linked Immunosorbent Assay (ELISA)

Blood samples from patients were placed at room temperature for 30 min, and then centrifuged at 3000 rpm for 15 min at 4 °C GDF3 in the collected sera was measured by ELISA kits purchased from Elabscience Biotechnology Co., Ltd. (Wuhan, China) according to the manufacturer’s instructions. At 12h post-LPS injection i.p. (10 mg/kg) mice, whole blood samples were collected by cardiac puncture using heparinized needles, and centrifuged at 12,000 rpm for 15 min to collect sera. Cell culture supernatants were harvested at 12 h after LPS treatment (10 ng/mL). The levels of TNF-α, IL-6, IL-1β and MCP-1 were determined, using ELISA kits purchased from BioLegend according to manufacturer’s instructions. Cytokine levels were established by comparison to a standard curve per the manufacturer’s instructions.

### 2.10. Statistical Analysis

Data were analyzed using the Statistical Package for the Social Sciences (SPSS version 19.0) or GraphPad Prism 7 (GraphPad Software, San Diego, CA, USA). Frequency and percentages were calculated for counted data and analyzed by chi-square (χ^2^) test. Median with interquartile ranges (IQR) was computed for a continuous variable and compared between indicated groups by independent *t*-test or nonparametric Mann-Whitney U test if the markers did not meet criteria for Gaussian distribution. Spearman rank-order correlations were applied to the correlation between GDF3 levels and APACHE II scores and SOFA scores. To evaluate discriminative values, we calculated the areas under the receiver operating characteristic curves (AUROCs) for variables significantly differing between sepsis and control groups, between non-survivors and survivor groups. Data from mice and cell experiments were presented as means ± SD and statistically analyzed using the Student’s *t* test. *p* < 0.05 was considered statistically significant. Survival is presented as Kaplan-Meier curves and differences were analyzed by the log-rank test in GraphPad Prism software.

### 2.11. Study Approval

Ethical approval for recruitment of septic patients and blood sample collection was granted by Clinical Research Ethics Committee, Renmin Hospital of Wuhan University (WDRY2019-K027) in conformity to the principles of the Declaration of Helsinki.

## 3. Results

### 3.1. Serum GDF3 Levels Are Higher in Septic Patients than Healthy Controls

**Figure 1:**
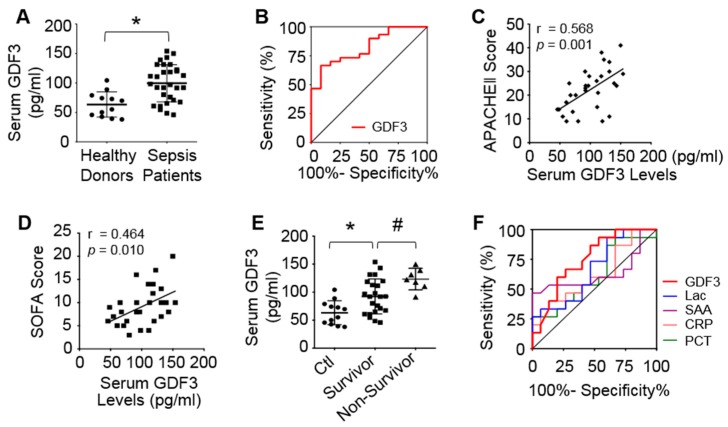
Higher serum levels of GDF3 are related to poor outcomes of septic patients. (**A**) Scatter plots of serum GDF3 levels on admission of healthy donors and septic patients. (**B**) AUC discriminating sepsis from healthy controls. (**C**,**D**) Correlation between serum GDF3 levels and (**C**) APACHE II score and (**D**) SOFA score at admission of ICU. (**E**) Serum GDF3 levels in different groups sorted by survivor and non-survivor. (**F**) AUROC predicting 28-day mortality: GDF3 (AUC 0.770), Lac (AUC 0.767), CRP (AUC 0.632), PCT (AUC 0.677), SAA (AUC 0.724). (*, *p* < 0.05 vs. healthy donors; #, *p* < 0.05 vs. survivor).

On admission, human patients with sepsis exhibited significantly higher serum GDF3 levels compared with healthy donors (Figure 1A). Interestingly, median GDF3 level of septic shock group was much higher than non-shock sepsis group (Supplementary Table S1). Furthermore, receiver operating characteristic (ROC) curves were generated for GDF3 to discriminate sepsis from healthy donors (Figure 1B). The area under the curve (AUC) of GDF3 was 0.825 with 95% confidence interval (CI, 0.697–0.953, *p* = 0.001), and optimal cut-off level was 90.23pg/mL (Figure 1B). Noteworthily, a positive correlation was observed between GDF3 levels and APACHEII score (r = 0.568, *p* = 0.001), or SOFA score (r = 0.464, *p* = 0.010) (Figure 1C,D), indicating that high serum GDF3 levels may correlate with severe multiple organ dysfunction. We also investigated whether GDF3 levels could predict 28-day mortality, and observed that higher median levels of serum GDF3 on admission correlate to higher mortality at 28-day follow-up, compared to survivor-group (122.50 pg/mL vs. 92.00 pg/mL, *p* = 0.031) (Figure 1E). To further clarify the capacity to predict mortality by serum GDF3 levels in sepsis patients, we conducted an ROC curve analysis. GDF3 levels showed stronger predictive capacity with AUC value of 0.770 (95% CI, 0.593-0.947, *p* = 0.033), when compared to other conventional sepsis biomarkers (i.e., lactate (Lac), C-reaction protein (CRP), procalcitonin (PCT) and serum amyloid A (SAA)) (Figure 1F and Supplementary Table S3). Put together, these pilot data suggest that higher serum levels of GDF3 are strongly associated with severity and mortality of septic patients.

### 3.2. The Expression of GDF3 Is Markedly Altered in the Blood, Spleen and Macrophages of Mice upon Endotoxin Challenge

**Figure 2:**
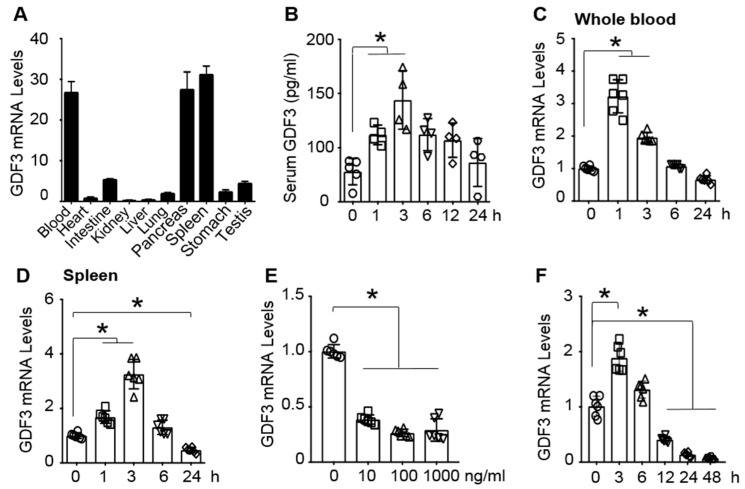
The dynamic expression of GDF3 in mice and macrophages treated with endotoxin. (**A**) mRNA levels of GDF3 in different tissues of mice, *n* = 4. GAPDH gene expression was used as the internal control. (**B**) Serum GDF3 levels are detected at indicated time points in mice after LPS (10mg/kg) injection (*, *p* < 0.05; *n* = 4). mRNA levels of GDF3 were determined in (**C**) whole blood and (**D**) spleens of LPS-injected mice (*, *p* < 0.05; *n* = 6). (**E**) LPS-dose response and (**F**) time course of GDF3 expression in BMDMs, where indicated, (**E**) total RNAs were collected at 24 h after LPS exposure (*, *p* < 0.05) and (**F**) BMDMs were pre-treated with LPS (10 ng/mL) (*, *p* < 0.05; *n* = 4). Similar results were obtained in other two independent experiments.

Previously, GDF3 has been shown to be highly expressed in spleens of human [17], implicating its potential effects on the immune system. Consistently, we observed that GDF3 was highly expressed in the spleen, the blood, and the pancreas, compared to other tissues of mice (Figure 2A). To explore the possible role of GDF3 in endotoxin-induced inflammation and organ failure, in particular, cardiac dysfunction, we determined the alterations of GDF3 expression in the blood and spleen of mice upon LPS challenge. Compared with 0 h, serum GDF3 levels were significantly elevated in mice at 1, 3 and 6 h after LPS injection (Figure 2B). Similarly, mRNA levels of GDF3 in whole blood and spleen of endotoxin-treated mice were greatly upregulated at 1 and 3 h, compared to basal conditions (Figure 2B–D). Of interest, GDF3 expression was significantly down-regulated in the spleen of mice at 24 h post-LPS injection, compared to 0-h controls (Figure 2D). Given that splenic macrophages are critical in dealing with blood-spread infection and activation of immune responses [18], we lastly focused on evaluating GDF3 expression in macrophages upon LPS challenge. BMDMs were treated with different doses of LPS for 24 h, and displayed remarkably decreased expression of GDF3 in response to different doses of LPS (Figure 2E). We then selected 10 ng/mL concentration of LPS to determine the time course of GDF3 expression in BMDMs, and observed that GDF3 mRNA level peaked at 3 h after LPS treatment and gradually decreased at later time points (Figure 2F). These results indicate that the expression of GDF3 is dynamically altered in macrophages, whole blood and spleen of mice upon endotoxin insult.

### 3.3. GDF3 Attenuates Endotoxin-Triggered Inflammatory Cytokine Production in Macrophages

**Figure 3:**
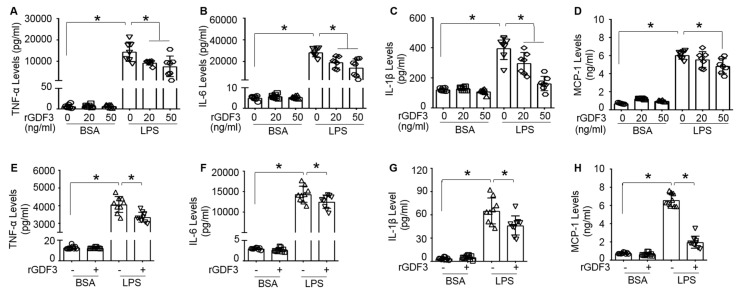
Recombinant GDF3 protein suppresses pro-inflammatory cytokine production in LPS-treated macrophages. (**A**–**D**) BMDMs treated with the indicated doses of recombinant GDF3 (rGDF3) and (**E**–**H**) RAW264.7 macrophages treated with rGDF3 (50 ng/mL) for 2 h, followed by addition of LPS (10 ng/mL) for 24 h. Culture supernatants were measured for (**A**,**E**) TNF-α, (**B**,**F**) IL-6, (**C**,**G**) IL-1β and (**D**,**H**) MCP-1 by ELISA (*, *p* < 0.05; *n* = 7–9 wells). Similar results were obtained in another separated experiments.

Considering the established link between macrophages and endotoxin-induced sepsis, and the vital role of macrophages in producing pro-inflammatory cytokines and mediating down-stream inflammation responses to infection [5], we then investigated whether GDF3 could affect the production of inflammatory cytokines in macrophages upon LPS stimulation. Cultured BMDMs were treated with different doses of rGDF3 or control BSA for 2 h, followed by addition of LPS (10 ng/mL) for 24 h. Cell culture supernatants were collected to measure TNF-α, IL-6, IL-1β and MCP-1 levels. Our ELISA results showed that LPS-induced productions of inflammatory cytokines were significantly suppressed by rGDF3, compared to BSA-controls (Figure 3A–D). Similar results were also observed in RAW264.7, a mouse macrophage cell line, when treated with rGDF3 (50 ng/mL) followed by LPS insult (Figure 3E–H). These results suggest that GDF3 could attenuate pro-inflammatory cytokine production from macrophages in response to endotoxin challenge.

### 3.4. GDF3 Inhibits LPS-Mediated Macrophage Pro-Inflammatory Polarization In Vitro

**Figure 4:**
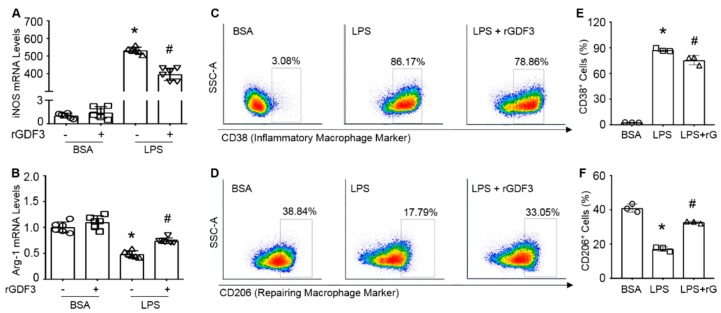
Recombinant GDF3 protein reduces macrophage M1 and increases M2 polarization in LPS-treated BMDMs. BMDMs were treated with rGDF3 (50 ng/mL) for 2 h, then added LPS (10 ng/mL) for 6 h. mRNA levels of (**A**) iNOS and (**B**) Arg-1 were evaluated by RT-PCR. The mRNA levels were normalized to 18S mRNA levels and expressed as fold versus BSA group (*, *p* < 0.05 vs. Ctl; #, *p* < 0.05 vs. LPS; *n* = 6). (**C**,**D**) BMDMs were treated with rGDF3 (50 ng/mL) for 2 h, followed by LPS (10 ng/mL) exposure for 12 h. These BMDMs were stained with antibodies to (**C**) CD38 and (**D**) CD206 for flow cytometry analysis. Quantification results for (**E**) CD38 positive macrophages and (**F**) CD206 positive macrophages. (*, *p* < 0.05 vs. BSA; #, *p* < 0.05 vs. LPS; *n* = 3). Similar results were obtained in other two separated experiments.

Given the reduced pro-inflammatory cytokine secretion mediated by rGDF3, we next tested whether GDF3 influenced macrophage phenotype. RT-PCR analysis of macrophage polarization markers (i.e., iNOS for M1 and Arg1 for M2) showed that LPS stimulation remarkably activated iNOS expression, whereas greatly inhibited Arg1 expression in BMDMs (Figure 4A,B), which are consistent with previous reports [19]. 

However, treatment of LPS-macrophages with rGDF3 resulted in a significantly reduction of iNOS expression (Figure 4A) and elevation of Arg-1 mRNA levels (Figure 4B), compared to control cells. We further performed flow cytometry analysis for cell surface markers of macrophage polarization (CD38 for M1, and CD206 for M2) in rGDF-treated BMDMs upon LPS challenge (Figure 4C,D). Consistent with mRNA expression data of iNOS (M1) and Arg1 (M2), the results of flow cytometry analysis also showed that rGDF3 significantly reduced the proportion of CD38+ BMDMs (M1, Figure 4C,E) and enhanced CD206+ BMDMs (M2, Figure 4D,F) in response to LPS insult (Figure 4C–F). It is important to note here, under basal conditions, rGDF3 did not influence BMDM phenotype after fully differentiation from bone marrow stem cells (Supplementary Figure S1). Together, both sets of experimental data consistently indicate that GDF3 could suppress LPS-induced pro-inflammatory macrophage M1 phenotype and promote M2 polarization in vitro.

### 3.5. GDF3 Suppresses Inflammatory Response and Improves Cardiac Function and Survival through Enhanced Cardiac M2 Macrophages in Endotoxin-Induced Septic Mice

**Figure 5:**
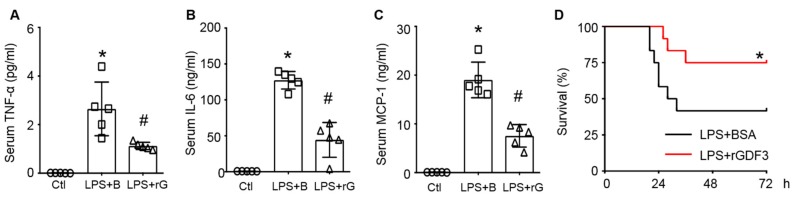
Recombinant GDF3 protein attenuates inflammatory response and mortality in endotoxin-induced septic mice. WT mice injected with rGDF3 protein (I.P., 10μg/kg BW) 12 h prior to LPS injection (I.P., 10mg/kg BW), 12 h later serum was collected for measuring (**A**) TNF-α, (**B**) IL-6, and (**C**) MCP-1 levels (*, *p* < 0.05 vs. Ctl; #, *p* < 0.05 vs. LPS + B; *n* = 5). (**D**) Kaplan-Meier survival curves were generated for LPS-mice treated with or without rGDF3 (*, *p* < 0.05; *n* = 12).

Considering that GDF3 could drive macrophages to M2 phenotype and suppress LPS-induced inflammation in vitro, we next sought to determine whether GDF3 had a similar effect in vivo. Hence, wild-type mice were administered with rGDF3 (10 μg/kg) or the same dose of BSA 12 h prior to LPS injection, mouse sera were collected for cytokine assays 12 h after LPS injection. ELISA results showed that serum levels of TNF-α, IL-6, and MCP-1 both were significantly increased in LPS-treated mice, compared with controls (Figure 5A–C), which are consistent with previous reports [20]. However, pre-administration of mice with rGDF3 remarkably decreased serum levels of TNF-α, IL-6, and MCP-1 upon LPS challenge (Figure 5A–C). Furthermore, Kaplan-Meier survival curves showed that mouse survival rate of LPS + rGDF3 group was dramatically higher than LPS-group (*p* = 0.043, Figure 5D).

**Figure 6:**
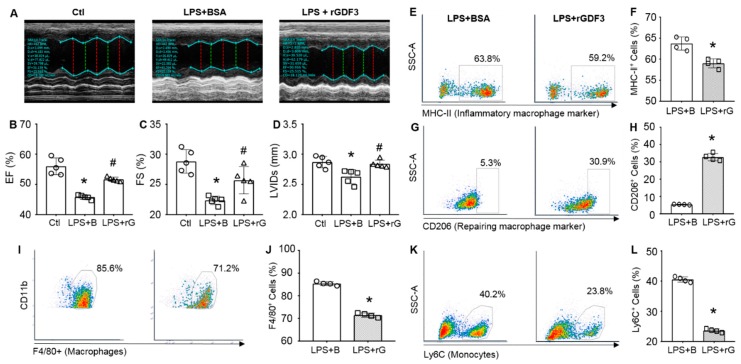
Administration of rGDF3 to LPS-mice improves cardiac function and promotes cardiac macrophage M2 phenotype. WT mice received recombinant GDF3 protein (I.P., 10μg/kg BW) 12 h prior to LPS injection (I.P., 10mg/kg BW), 12 h later cardiac function was measured by echocardiograph. (**A**) Representative M-mode echocardiography recordings for three groups, and (**B**) left ventricular ejection fraction (EF %), (**C**) fractional shortening (FS %) and (**D**) left ventricular internal dimension at end-systolic (LVIDs) were calculated (*, *p* < 0.05 vs. Ctl; #, *p* < 0.05 vs. LPS+B; *n* = 5). Cardiac cells were stained with antibodies to (**E**) MHC-II, (**G**) CD206, (**I**) F4/80 plus CD11b, (**K**) Ly6C for flow cytometry analysis and their respective quantification results are shown in (**F**,**H**,**J**,**L**). (*, *p* < 0.05; *n* = 4). Similar results were obtained in other two independent experiments.

Currently, it is well recognized that cardiac dysfunction represents the major cause of death in sepsis [4]. We then assessed the effect of rGDF3 on cardiac function in LPS-induced septic mice by echocardiography (Figure 6A). As shown in Figure 6A–D and Supplementary Table S4, pre-injection of rGDF3 in LPS-mice markedly improved myocardial contractile function, as evidenced by higher EF % (Figure 6B), FS % (Figure 6C), and LVIDs (Figure 6D), when comparing with BSA-treated group.

Given that LPS could stimulate infiltration of monocytes/macrophages into the heart where significantly influence contractile function [21], we thus determined whether rGDF3 affected cardiac macrophage phenotype in LPS-treated mice. As shown in Figure 6E,F,K,L, the percentage of cardiac MHC-II+ and Ly6C+ macrophages (pro-inflammatory macrophages, M1) was significantly reduced in LPS + rGDF3 group, compared to control LPS + BSA group. In contrast, the percentage of CD206-positive macrophages (repairing anti-inflammatory macrophages, M2) was dramatically increased in rGDF3-treated mice upon LPS challenge, compared to BSA-treated control mice (Figure 6G/H). Notably, recombinant GDF3 also prevented LPS-triggered infiltration of macrophages (F4/80+CD11b+ cells) (Figure 6I,J) and monocytes (Ly6C+) (Figure 6K,L) into hearts. Taken together, these in vivo data suggest that GDF3 not only suppresses LPS-induced systemic inflammation, but also reduces monocyte/macrophage migration to hearts of mice after LPS stimulation and more importantly, facilitates M2 macrophage polarization, leading to improved cardiac function and animal survival.

### 3.6. GDF3 Activates Smad2/3 Signaling to Drive Macrophage M2 Polarization

**Figure 7:**
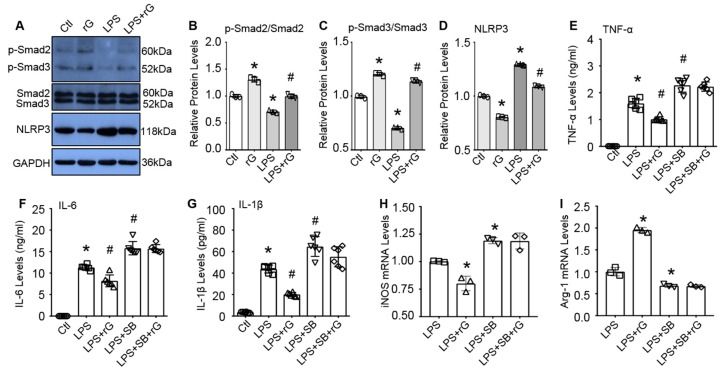
GDF3-mediated macrophage M2 phenotype is associated with the activation of Samd2/3 and inhibition of NLRP3 inflammasome. (**A**) Representative immuno-blots and quantification results for (**B**) p-Samd2, (**C**) p-Smad3 and (**D**) NLRP3. GAPDH was used as a loading control for total protein (*, *p* < 0.05 vs. Ctl cells, #, *p* < 0.05 vs. LPS treated cells; *n* = 3). (**E**–**G**) RAW264.7 macrophages were pre-treated with SB431542 or DMSO (10μM) for 0.5 h, then incubated with rGDF3 (50 ng/mL) or BSA for 1 h, followed by stimulation with LPS (10 ng/mL) for 12 h. Supernatants were collected to measure (**E**) TNF-α, (**F**) IL-6, and (**G**) IL-1β levels (*, *p* < 0.05 vs. Ctl cells; #, *p* < 0.05 vs. LPS-treated cells; *n* = 4). The mRNA levels of (**H**) iNOS and (**I**) Arg-1 in these macrophages treated as above were measured by RT-PCR. The fold changes were normalized to 18S (*, *p* < 0.05 vs. LPS-cells; *n* = 4). Data shown were representative of three independent experiments.

Lastly, we sought to elucidate mechanisms underlying GDF3-mediated M2 macrophage polarization and suppression of inflammatory response to endotoxin. Previous studies have shown that GDF3 could activate ALK4/5/7 and their downstream Smad2/3 signaling in adipose tissue, skeletal muscle [8,10,22]. In addition, activation of Smad2/3 is able to downregulate NLRP3-inflammasome expression in colon tissue [23]. Hence, we hypothesized that GDF3 may activate Smad2/3 signaling cascade in macrophages and thereby, promote M2 polarization and suppress LPS-triggered inflammation. To test this hypothesis, we pre-treated RAW264.7 macrophages with or without rGDF3 (50 ng/mL) for 12 h, followed by addition of LPS (10 ng/mL) for 3h. Then these cells were collected to isolate total protein for western blot analysis. As shown in Figure 7A–C, treatment of macrophages with rGDF3 resulted in significantly higher levels of phosphorylated Smad2/3, compared to control cells. By contrast, LPS treatment yielded significantly lower levels of phosphorylated Smad2/3, when comparing to control cells. However, LPS-caused reductions of phosphorylated Smad2/3 were greatly inhibited by addition of rGDF3 (Figure 7A–C). Accordingly, protein levels of NLRP3 were significantly decreased in rGDF3-treated cells, whereas being greatly increased in LPS-treated cells, compared to controls. Similarly, LPS-induced elevation of NLRP3 protein levels in macrophages was significantly attenuated by treatment with rGDF3 (Figure 7A,D). These data indicate that exogenous GDF3 is able to activate Smad2/3 signaling pathway in macrophages.

To further determine whether GDF3-mediated suppression of LPS-induced inflammation and M2 polarization are dependent on the Smad2/3 activation, we pre-treated RAW264.7 macrophages with or without SB431542 (10μM), a specific inhibitor of Smad2/3, for 30 min, then incubated with rGDF3 (50 ng/mL) or BSA for 1 h, followed by stimulation with LPS (10 ng/mL) for 12 h. Cell supernatants were harvested to measure pro-inflammatory cytokine levels, using ELISA kits. As shown in Figure 7E–G, LPS-stimulated releases of TNF-α, IL-6, and IL-1β by macrophages were significantly inhibited by addition of rGDF3. However, such rGDF3-elicted inhibitory effects in LPS-macrophages were greatly offset by addition of SB431542, as evidenced by failure of exogenous GDF3 to reduce the levels of TNF-α, IL-6, and IL-1β in LPS-macrophages when pre-treated with SB431542 (Figure 7E–G). Moreover, expression levels of iNOS (M1 marker) were remarkably decreased, whereas mRNA levels of Arg1 (M2 marker) were dramatically increased in rGDF3-treated LPS-cells, compared to LPS-controls. When pre-addition of SB431542, however, such rGDF3-induced changes of macrophage phenotype were not observed (Figure 7H,I). Thus, these data suggest that GDF3 signaling through the Smad2/3-NLRP3 pathway is critical for macrophage M2 polarization with the associated inhibitory effects on endotoxin-caused inflammation.

## 4. Discussion

Sepsis is initiated by an infection, and the absence of intrinsic anti-inflammatory signaling at the early stage can cause an exaggerated activation of immune cells, including monocytes and macrophages, leading to hyper-inflammatory “cytokine storm” and subsequently contributes to organ dysfunction. Emerging evidence has shown that macrophage phenotypic alterations play a pivotal role during sepsis [5]. In general, M1 macrophages are pro-inflammatory; their apoptosis during the process of pathogen clearance simultaneously dampens the inflammatory response and transition of M1 to tissue-repairing M2 macrophages [7,24]. Therefore, understanding the mechanisms that control macrophage polarization would be crucial for the improvement of immune therapy against sepsis.

In the present study, we identified and described the dynamic changes of GDF3 in the blood and the spleen of mice upon endotoxin LPS challenge as well as LPS-treated macrophages, which exhibited increases at the early stage and decreases at the later stage. More interestingly, serum levels of GDF3 were greatly elevated in human patients with severe sepsis on admission, which implicates its potential compensatory role in host immune response. Indeed, treatment of macrophages with recombinant GDF3 protein strongly facilitated M2 macrophages transition, compared to controls cells cultured in the presence of LPS. Similarly, in vivo administration of endotoxin-induced septic mice with recombinant GDF3 protein significantly enhanced macrophage M2 polarization in the heart, leading to improved cardiac function and animal survival. Consistent with our findings presented here, Arora et al recently demonstrated that the ATP-binding cassette gene ABCF1 drives macrophages to an M2 phenotype and consequently, protects mice against endotoxin-induced sepsis mortality [25]. Tang et al further showed that adoptive transfer of ex vivo programed M2 macrophages into endotoxin-induced septic mice significantly increased the survival rate [26]. These results indicate that the modulation of macrophages to M2 phenotype might be an appropriate strategy for treatment of sepsis. However, caution has to be taken with regards to specific roles of each macrophage phenotype during sepsis, because a couple of recent studies also reported that macrophage polarization into M1 phenotype improved organ dysfunction and reduced mortality in lethal sepsis through enhanced bacterial phagocytosis and killing. Controversially, Taratummarat et al observed that gold nanoparticles (AuNP) administration at the time of CLP (cecal ligation and puncture) improved the survival and decreased bacterial burdens though the induction of macrophage polarization toward anti-inflammatory (M2) [27]. Thus, an intricate balance of the pro-inflammatory M1 and the tissue-repairing M2 macrophages, as well as sufficient transition toward M2 phenotype at later stage, would be critical to improve sepsis survival.

**Figure 8:**
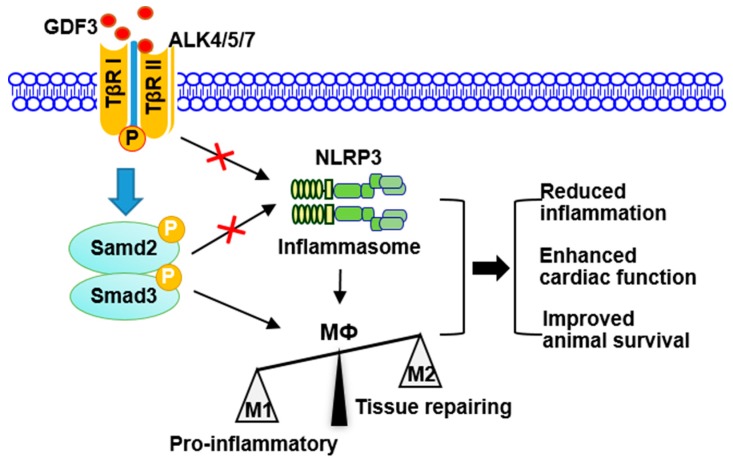
Scheme depicting GDF3-drived protection against endotoxin-induced inflammation, cardiac dysfunction and mortality. Exogenous GDF3 binds to ALK4/5/7 where activates Smad2/3 and inhibits NLRP3 inflammasome, consequently suppresses macrophage pro-inflammatory phenotype (M1), leading to reduced inflammation, cardiac dysfunction, and mortality in endotoxin-induced septic mice.

As for how GDF3 regulates macrophage M2 polarization, our data presented in this study suggest that exogenous GDF3 could activate Smad2/3-NLRP3 signaling through binding to TGF-β receptors ALK4/5/7 in macrophages, leading to M2 phenotype. This is evidenced by that: (1) increased levels of phosphorylated Smad2/3 in GDF3-treated macrophages with or without LPS insult; and (2) blockade of ALK4/5/7 with a specific inhibitor SB431542 dramatically suppressed GDF3-mediated elevation of Arg1 (M2 marker). In accordance with our findings, Sugiyama et al reported that Smad2 and Smad3 negatively regulate iNOS (M1 marker) expression in tumor-associated macrophages [28]. Additionally, mice lacking TGFβ type II receptor (TβRII), an upstream kinase of Smad2/3, develop a lethal inflammatory through the impaired macrophage M2 polarization [29,30]. Furthermore, there is emerging evidence that GDF11-mediated activation of Smad2/3 could inhibit the expression of NLRP3 inflammasome in the colon [23]. Currently, it has been shown that NLRP3 inflammasome could modulate macrophages to M1 phenotype [31,32]. Therefore, these prior studies, together with our present work, consistently indicate that GDF3-drived macrophage M2 polarization could be largely through the activation of Smad2/3 and the suppression of NLRP3 inflammasome, leading to reduced inflammation, cardiac injury and mortality in endotoxin-induced septic mice (Figure 8). In addition to M2 polarization, administration of recombinant GDF3 protein into endotoxin-induced septic mice also significantly reduced the infiltration of monocytes/macrophages into hearts, compared to control samples. This could be attributed to the reduced expression of monocyte chemoattractant protein (MCP)-1, a potent factor for monocyte and macrophage migration and infiltration, while the detailed underlying mechanism remains to be clarified [33].

Lastly, our data generated from a mouse model are seemingly at odds with clinical data showing the significantly increased serum levels of GDF3 in non-survival septic patients. Given the complexity of sepsis pathophysiology [34,35] and incomplete knowledge of mediators that drive mortality, the reasons for these discrepancies are unclear but could reflect dual roles of GDF3 in sepsis patho-physiology. At the early inflammatory stage (i.e., endotoxic shock model), acute administration of GDF3 into endotoxic mice could increase survival outcome and improve cardiac function through anti-inflammatory response by suppression of macrophage M1 polarization. However, in human sepsis patients, constitutive high levels of GDF3 might be associated with lethality because GDF3 could promote macrophage polarization toward M2 phenotype which leads to immunosuppression. Similar findings are also observed in other sepsis mediators. For example, human patients that died during the course of sepsis treatment had significantly higher serum GDF15 levels on admittance to the ICU than survivors [36]. However, a recent study by Luan et al demonstrated that exogenous administration of GDF15 well after the initial inflammatory phase of septic mouse model is sufficient to improve survival rate by modulating systemic levels of plasma triglycerides [37]. Another case similar to our study is lactate which exhibits higher levels in septic shock patients [38]. It is well known that serum lactate levels are served as a clinical marker of sepsis severity [39]. Nonetheless, Colegio et al reported that lactate could promote macrophage M2 polarization and inhibit sepsis-induced inflammation [40]. There are several limitations to this study. First, the present clinical data just provide a pilot observation due to small sample size. Whether GDF3 could serve as a clinical marker of sepsis/septic shock remains to be further validated using a large cohort of sepsis patient samples. Second, while endotoxin is a major causative factor for gram-negative bacteria-induced sepsis [41], it will however be interesting to investigate if GDF3 confers similar protection against true microbe-induced sepsis in the future.

## 5. Conclusions

In conclusion, GDF3 may represent a new biomarker candidate for sepsis that could predict mortality and severity. Both in vitro and in vivo data show that GDF3 could suppress macrophage M1 polarization and inhibit endotoxin-induced inflammatory response (Figure 8). Acute administration of recombinant GDF3 protein in septic mice protects against endotoxin-induced cardiac dysfunction and sepsis mortality. Mechanistically, we identify that GDF3-mediated protection is associated with activation of Smad2/3 and suppression of NLRP3 inflammasome in macrophages (Figure 8). Thus, GDF3 may serve as a new supplement to immunotherapy treatment of sepsis from a translational viewpoint, and as a potential novel biomarker for the diagnosis of sepsis.

## Supplementary Materials

The following are available online at https://www.mdpi.com/2073-4409/9/1/120/s1, Table S1: Demographics and clinical details about non-shock sepsis; Table S2. The primer sequences for RT-PCR; Table S3. AUC analysis for 28-day mortality prediction within the derivation; Figure S1. GDF3 does not affect BMDM phenotype after fully differentiation from bone-marrow stem cells; Table S4. Echocardiographic analysis for cardiac function in LPS-mice treated with or without rGDF3.
