# Supplementary material for: GDF3 Protects Mice against Sepsis-Induced Cardiac Dysfunction and Mortality by Suppression of Macrophage Pro-Inflammatory Phenotype
Source: Cells. 2020 Jan 3;9(1):120. doi: 10.3390/cells9010120 (PMC7017037; doi:10.3390/cells9010120)
Supplement: Supplementary file 1 [file cells-09-00120-s001.pdf]

Supplementary Information

Table S1. Demographics and clinical details about non-shock sepsis and septic shock patients

Variables	Total Patients (n = 30)	Non-shock Sepsis (n = 15)	Septic Shock (n = 15)	*p* value
Age (y), median (IQR)	63 (54-71)	65 (59-71)	60 (51-71)	0.350
Gender, male, n (%)	17 (56.7%)	7 (46.7%)	10 (66.7%)	0.269
Death before Day 28, n (%)	7 (2.3%)	0	7 (46.7%)	0.003*
BMI, kg/m^2, median (IQR)	25 (22-27)	25 (23-27)	25 (22-27)	0.719
Hospital length of stay (days), median (IQR)	9 (9-10)	9 (9-10)	9 (9-10)	0.585
APACHE Ⅱ score, median (IQR)	23.5 (14.3-28)	14 (11-21)	28 (25-32)	< 0.001*
SOFA score, median (IQR)	8 (6-10)	6 (5-8)	10 (10-14)	< 0.001*
**Hematologic parameters**				
Hemoglobin, g/L, median (IQR)	100.5 (86.8-115)	95 (88-108)	101 (88-119)	0.455
White Blood Cells×10^9/L, median (IQR)	13.1 (8.7-25.2)	12.8 (10.1-22.9)	13.3 (7.7-26.0)	0.828
Neutrophils×10^9/L, median (IQR)	12.1 (7.4-25.8)	12.0 (8.9-26.8)	12.3 (6.4-24.5)	0.342
Platelets×10^9/L, median (IQR)	89.0 (45.5-145.0)	109.0 (65.0-142.0)	85.0 (35.0-131.0)	0.385
**Metabolic and Coagulation biomarkers**				
Albumin, g/L, median (IQR)	29.8 (27.9-31.8)	30.0 (29.0-32.0)	29.0 (26.2-31.3)	0.455
Lactate, mmol/L, median (IQR)	2.7 (2-5)	2.3 (1.2-3.7)	3.3 (2.4-7.5)	0.038*
Creatinine, μmol/L, median (IQR)	170.5 (90.5-275.0)	158.0 (74.5-332.5)	172.0 (133.0-268.5)	0.767
Glucose, mmol/L, median (IQR)	7.3 (6.5-10.2)	7.4 (6.6-10.2)	7.1 (6.5-10.1)	0.587
APTT, sec, median (IQR)	37.4 (32.0-40.1)	33.8 (29.8-38.4)	39.1 (36.8-41.0)	0.057
**Classic biomarkers of sepsis**				
CRP, mg/L, median (IQR)	156.4 (64.7-235.8)	115.0 (54.2-197.1)	171.1 (72.4-273.0)	0.264
PCT, ng/mL, median (IQR)	40.0 (8.6-101.5)	35.9 (4.7-90.8)	48.5 (14.1-154.0)	0.300
SAA, mg/L, median (IQR)	244.5 (147.9-275.3)	234.0 (199.5-265.0)	267.0 (127.5-429.5)	0.229
GDF3, pg/ml, median (IQR)	97.5 (70.29-124.70)	80.0 (60.53-111.50)	118.1 (92.00-131.30)	0.019*

APACHE II score, acute physiology, age and chronic health evaluation II score; SOFA score, sequential organ failure assessment score; APTT, activated partial thromboplastin time; CRP, C-reaction protein; PCT, procalcitonin; SAA, serum amyloid A; GDF3, growth differentiated factor 3. (*, $p < 0.05$ shock *vs.* non-shock sepsis group)

Table S2. The primer sequences for RT-PCR

	Forward	Reverse
GDF3	CTATAACCTGCGACCAGAGCTG	CAGTCGGTTGCTGCTCCAATCC
iNOS	CAGCTGGGCTGTACAAACCTT	CATTGGAAGTGAAGCGTTTCG
Arg1	TCATGGAAGTGAACCCAACTCTTG	TCAGTCCCTGGCTTATGGTTACC
18S	GTTCCGACCATAAACGATGCC	TGGTGGTGCCCTTCCGTCAAT
GAPDH	TCTGACGTGCCGCCTGGAGA	CAGCCCCGGCATCGAAGGTG

Table S3. AUC analysis for 28-day mortality prediction within the derivation

	AUROC	95%CI	*p* value	Optimal Cut-off	Sensitivity (%)	Specificity (%)	PPV (%)	NPV (%)
GDF3	0.770	0.593-0.947	0.030	104.40	85.71	65.22	85.71	65.22
Lac	0.767	0.553-0.982	0.035	6.175	57.14	91.3	66.67	87.50
CRP	0.632	0.471-0.792	0.097	260.5	42.86	92.11	66.67	87.50
PCT	0.677	0.460-0.894	0.162	9.200	100	34.78	31.82	100.00
SAA	0.724	0.472-0.975	0.078	311.5	57.14	86.96	57.14	86.96

GDF3, growth differentiated factor 3; Lac, lactate; CRP, C-reaction protein; PCT, procalcitonin; SAA, serum amyloid A.

Figure S1.

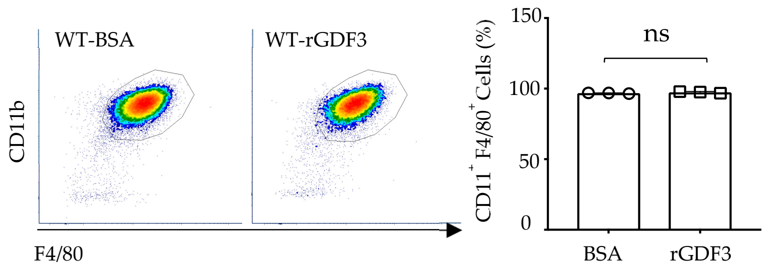

Figure S1. GDF3 does not affect BMDM phenotype after fully differentiation from bone marrow stem cells. Purity of BMDMS showed no difference after incubation with rGDF3 (50ng/ml, 2 h), followed by LPS treatment (10ng/ml, 12 h), when compared to BSA-treated control group. BMDMs were stained with antibodies to CD11b and F4/80 for flow cytometry analysis (n=3 independent experiments).

Table S4. Echocardiographic analysis for cardiac function in LPS-mice treated with or without rGDF3

Parameter	Ctl (n = 5)	LPS (n = 5)	LPS+rGDF3 (n = 5)
LVIDs (mm)	2.869±0.094	2.627±0.115*	2.832±0.034*#
LVIDd (mm)	4.034±0.033	3.382±0.150*	3.554±0.033*#
Vs	31.48±2.49	25.46±2.51*	29.41±0.98*#
Vd	71.49±1.37	47.00±4.89*	52.81±1.20*#
SV	41.01±1.43	21.54±2.41*	24.88±0.81*#
EF (%)	56.00±2.76	45.81±0.72*	51.71±0.72*#
FS (%)	28.8±1.94	22.36±0.69*	25.68±2.27*#

LVIDs, left ventricular end-systolic inner diameter; LVIDd, left ventricular end-diastolic inner diameter; Vs, end-systolic volume; Vd, end-diastolic volume; SV, stroke volume; EF, ejection fraction ; FS, fractional shortening. (*, $p < 0.05$ *vs.* Ctl; #, $p < 0.05$ *vs.* LPS; n=5)
